# Lipid Metabolism, Oxidative Stress and Cell Death Are Regulated by PKC Delta in a Dietary Model of Nonalcoholic Steatohepatitis

**DOI:** 10.1371/journal.pone.0085848

**Published:** 2014-01-15

**Authors:** Michael W. Greene, Christine M. Burrington, Darin T. Lynch, Samantha K. Davenport, Andrew K. Johnson, Melissa J. Horsman, Saleem Chowdhry, Jian Zhang, Janet D. Sparks, Paul C. Tirrell

**Affiliations:** 1 Boshell Diabetes and Metabolic Disease Research Program, Auburn University, Auburn, Alabama, United States of America; 2 College of Human Sciences, Auburn University, Auburn, Alabama, United States of America; 3 Bassett Research Institute, Bassett Medical Center, Bassett Healthcare Network, Cooperstown, New York, United States of America; 4 Department of Pathology, Bassett Medical Center, Bassett Healthcare Network, Cooperstown, New York, United States of America; 5 Department of Internal Medicine, Bassett Medical Center, Bassett Healthcare Network, Cooperstown, New York, United States of America; 6 University of Rochester Medical Center, School of Medicine and Dentistry, Rochester, New York, United States of America; Bambino Gesu' Children Hospital, Italy

## Abstract

Steatosis, oxidative stress, and apoptosis underlie the development of nonalcoholic steatohepatitis (NASH). Protein kinase C delta (PKCδ) has been implicated in fatty liver disease and is activated in the methionine and choline-deficient (MCD) diet model of NASH, yet its pathophysiological importance towards steatohepatitis progression is uncertain. We therefore addressed the role of PKCδ in the development of steatosis, inflammation, oxidative stress, apoptosis, and fibrosis in an animal model of NASH. We fed PKCδ^−/−^ mice and wildtype littermates a control or MCD diet. PKCδ^−/−^ primary hepatocytes were used to evaluate the direct effects of fatty acids on hepatocyte lipid metabolism gene expression. A reduction in hepatic steatosis and triglyceride levels were observed between wildtype and PKCδ^−/−^ mice fed the MCD diet. The hepatic expression of key regulators of β-oxidation and plasma triglyceride metabolism was significantly reduced in PKCδ^−/−^ mice and changes in serum triglyceride were blocked in PKCδ^−/−^ mice. MCD diet-induced hepatic oxidative stress and hepatocyte apoptosis were reduced in PKCδ^−/−^ mice. MCD diet-induced NADPH oxidase activity and p47^phox^ membrane translocation were blunted and blocked, respectively, in PKCδ^−/−^ mice. Expression of pro-apoptotic genes and caspase 3 and 9 cleavage in the liver of MCD diet fed PKCδ^−/−^ mice were blunted and blocked, respectively. Surprisingly, no differences in MCD diet-induced fibrosis or pro-fibrotic gene expression were observed in 8 week MCD diet fed PKCδ^−/−^ mice. Our results suggest that PKCδ plays a role in key pathological features of fatty liver disease but not ultimately in fibrosis in the MCD diet model of NASH.

## Introduction

Non-alcoholic fatty liver disease (NAFLD) is characterized by the accumulation of lipids in the liver (steatosis) and may be a benign condition [1]. Its prevalence in the middle-aged segment of the population on a western diet is approximately 46% and of this group 30% are suggested to have non-alcoholic steatohepatitis (NASH) [2]. The prevalence of NAFLD in nonobese subjects has been reported to be 7.4% and 8.7% in the United States and India, respectively [3], [4]. The exact etiology for transformation of steatosis to NASH remains obscure; however, a classical “two-hit” hypothesis has been proposed to explain progression [5]. Steatosis constitutes the “first hit.” Proinflammatory cytokines (*e.g.* tumor necrosis factor-alpha, TNFα), oxidative stress, and lipid peroxidation constitute the “second hit” leading to NASH [1], [6]. Recently an alternative “non triglyceride lipotoxicity” hypothesis has been put forward implicating metabolites of free fatty acids in hepatocyte injury and development of NASH [7].

The classical (α, β, and γ) and novel (δ, ε, and θ) protein kinase C (PKC) isoforms are intracellular signaling molecules activated by lipids [8]. Lipid infusion activates muscle and hepatic novel PKC isoforms (PKCδ, PKCε, and PKCθ) but not that of classical or atypical PKC isoforms [9]–[11]. The PKCδ isoform can regulate lipid metabolism in the heart [12] and hepatic glucose production through a possible gut-brain-liver axis [13], suggesting a role for PKCδ in metabolic disease. Further, recent studies demonstrating that the PKCδ isoform regulates high fat diet-induced hepatic steatosis and the expression of hepatic lipogenic genes [14], [15] suggest that PKCδ plays an important role in lipid-associated liver disease.

Our recent studies in methionine and choline deficient (MCD) diet fed mice, which develop hepatic steatosis, inflammation, apoptosis, and fibrosis histologically similar to human NASH [16], [17], demonstrated that PKCδ protein expression and activation are elevated in the liver of mice fed the MCD diet compared to a control diet [18]. Furthermore, we observed in a cellular model of NASH that PKCδ knockdown blocked JNK activation and blunted palmitate-induced apoptosis [18]. In the present study, we questioned the role of PKCδ in regulating key pathophysiological features of NASH using the MCD diet model of NASH.

## Materials and Methods

### Animals

Heterozygous PKCδ^−/+^ mice in a mixed 129SX1×C57BL/6 background were backcrossed up to six times with C57BL/6NHsd mice from Harlan Laboratories (Somerville, NJ) and then interbred to generate PKCδ^−/−^ mice and wildtype littermates (WT). PKCδ genotyping was performed as previously described [19]. Mice were housed 2–4 per cage in Thoren units in the Bassett Research Institute, an AAALAC accredited animal facility, in light/dark (12L∶12D), temperature 22°C, and humidity controlled rooms. Mice were provided with standard laboratory chow and water ad libitum. Six to eight week old PKCδ^+/+^ and PKCδ^−/−^ mice (n = 6–8) were placed on a control or MCD diet (MP Biomedical, Cat #960441 or #960439, respectively) for four or eight weeks. No procedures were undertaken that caused more than minimal pain, distress, or discomfort. Mice were euthanized by inhalation of CO_2_. Blood and tissue samples were taken and processed as previously described [18]. This study was carried out in strict accordance with the recommendations in the Guide for the Care and Use of Laboratory Animals of the National Institutes of Health. The protocol was approved by the Mary Imogene Bassett Hospital Institutional Animal Care and Use Committee (Protocol Number: 11–36).

### Immunobloting and Antibodies

Frozen liver tissue was processed to generate total cell lysate extracts and membrane and cytosolic protein extracts as previously described [18], [20]. Polyclonal antibodies to phospho-PKCδ (Thr505), phospho-PKCδ (Ser643), phospho-PERK (Thr980), JNK1/2, caspase 9 (mouse specific), caspase 3, cleaved caspase 3 (Asp175), and monoclonal antibodies to phospho-JNK (Thr183/Tyr185) and IRE1α were from Cell Signaling Technology (Danvers, MA). Rabbit monoclonal antibodies to p67^phox^ and p91^phox^ (NOX2) were from Epitomics (Burlingame, CA). Polyclonal antibodies to p24^phox^ and p47^phox^ were from EMD Millipore (Billerica, MA).

Polyclonal antibodies to PKCδ (C-17), PKCε (C-15), PKCθ (C-18), PKCα (C-20), and p22^phox^ (FL-195) and monoclonal antibodies to GAPDH (6C5) were from Santa Cruz Biotechnology (Santa Cruz, CA). A polyclonal antibody to calnexin and monoclonal antibody to PKCδ were from Calbiochem/EMD Biosciences (La Jolla, CA). Monoclonal antibodies to αtubulin and BiP/GRP78 were from Sigma-Aldrich (St. Louis, MO) and BD Biosciences (San Jose, CA), respectively. Goat anti-mouse and anti-rabbit peroxidase conjugated antibodies were from Sigma (St. Louis, MO). ECL Plus from GE Healthcare was used for detection.

### Primary Hepatocyte Culture

Mice were anesthetized with an intraperitonal injection of pentobarbital (80 mg/kg). Perfusion was performed with well-oxygenated, calcium free Hank's buffer containing 5 mM glucose, 1.5 mM Na lactate, 0.15 mM Na pyruvate, 0.1 mM EGTA, 10 mM HEPES, 100 IU/ml Penicillin and 0.1 mg/ml Streptomycin maintained at 37°C, followed by perfusion with 0.05% collagenase H (Roche, Indianapolis, IN) in low-glucose DMEM (Life Technologies Corporation, Carlsbad, CA) containing 10 mM HEPES, 40 mM NaHCO_3_ and Pen/Strep. The hepatocytes were isolated from non-parenchymal cells using buffered Percoll and cultured in Waymouth's media (Life Technologies Corporation, Carlsbad, CA) containing 5% bovine growth serum for 2 h prior to treatment in serum free Waymouth's media for 18 h with BSA-complexes of 0.4 mM of palmitate or oleate or BSA prepared as previously described [18].

### Liver Tissue Histological and Lipid Analysis

Paraffin embedded sections were stained with hematoxylin and eosin or Masson's trichrome and then examined in a blinded fashion by a board certified pathologist, grading for steatosis, inflammation, and fibrosis as previously described [18]. Sirius Red staining of paraffin embedded sections was scored in a blind fashion using the METAVIR scoring system [21]. Lipids were extracted from approximately 100 mg of ground frozen liver tissue as described by Bligh and Dyer [22] Triglycerides were assayed using a kit from Thermo Scientific (Rockford, IL) and normalized to the protein content measured using the BCA protein assay reagent (Thermo Scientific/Pierce, Rockford, IL).

### Serum Metabolic Parameters

Alanine aminotransferase (ALT) and triglycerides were assayed as previously described [18]. Insulin was assayed using the Ultra Sensitive Mouse Insulin ELISA Kit from Crystal Chem Inc (Downers Grove, IL). NEFA was assayed using the kit from Zen-Bio, Inc. (Research Triangle Park, NC).

### Liver Oxidative Stress Analysis

Liver samples were flash frozen and ground in liquid nitrogen. Ground tissue (50–100 mg) was homogenized on ice in PBS pH 7.4 buffer. The homogenate was tested for thiobarbituric acid reactive substances (TBARS) (ZeptoMetrix, Buffalo, NY) following manufacturer's instructions. Protein content was determined using the Pierce BCA Protein assay (Thermo Scientific/Pierce, Rockford, IL). TBARS units (nmoles/ml) were normalized to protein concentration. 4-Hydroxy-2-nonenal (4-HNE) staining was performed using a 4-HNE (HNE11-S) antibody (Alpha Diagnostics, San Antonio, TX). Five random fields per slide were scored and the results were determined from an average of those scores. NADPH oxidase activity was measured by the lucigenin enhanced chemiluminescence method. Briefly, fifty micrograms of membrane protein fractionated from frozen liver as previously described [18] was added to Krebs-Ringer buffer, pH 7.0, containing 1 mM EGTA, 150 mM sucrose, 5 µM lucigenin, and 100 µM NADPH. Photon emission in terms of relative light units was measured in a luminometer every 30 s for 5 min. There was no measurable activity in the absence of NADPH. Superoxide anion production was expressed as relative chemiluminescence (light) units (RLU)/mg protein. Protein content was measured using the BCA protein assay reagent (Thermo Scientific/Pierce, Rockford, IL).

### Apoptosis Analysis

TUNEL positive cells were detected using the DeadEnd Fluorometric TUNEL system (Promega, Madison, WI) and the manufacturer's recommendations for controls. Propidium iodide (0.25 mg/ml) was used as the counterstain. An average score was generated based on a ratio of positive nuclei to total nuclei in 3 random fields.

### RNA isolation and qRT-PCR

TRIZOL reagent (Sigma-Aldrich, St. Louis, MO) was used to isolate total RNA from frozen liver tissue and cultured primary hepatocytes. RNA quantity and quality was assessed using a bioanalyzer (Agilent 2100 *Bioanalyze*, Agilent Technologies, Santa Clara, CA) prior to reverse transcription using the RT^2^ First Strand Kit (Qiagen, Valencia, CA). PCR was performed in 384 well plates with the RT^2^ SYBR Green ROX qPCR Mastermix (Qiagen, Valencia, CA) with gene specific primers (SABiosciences, Fredrick, MD) using an Applied Biosystems 7900HT Sequence Detection System (Life Technologies Corporation, Carlsbad, CA) with a Corbett Robotics CAS-1200 precision liquid handling system for plate set-up. Melting curve analysis was performed to verify product purity. Threshold values of 0.023 and 0.02 were used for the analysis of liver and hepatocyte gene expression, respectively. *GAPDH* and the geometric mean of *ACTB*, *GAPDH*, *GUSB*, and *HPRT* were used to normalize ΔCt values for hepatocyte and liver gene expression, respectively.

### Statistical analysis

All data are presented as the mean ±1 standard error (S.E.). Statistical significance was determined by Student's t-test or the Mann-Whitney Rank Sum test (α = 0.05) or a one- or two-way repeated measures analysis of variance (α = 0.05) using the XLSTAT 2009 program (Addinsoft, New York, NY). Pair-wise comparisons were made using Tukey's test (α = 0.05).

## Results

### Effect of MCD diet on body and organ weights, serum metabolites and PKCδ activation

**Figure 1:**
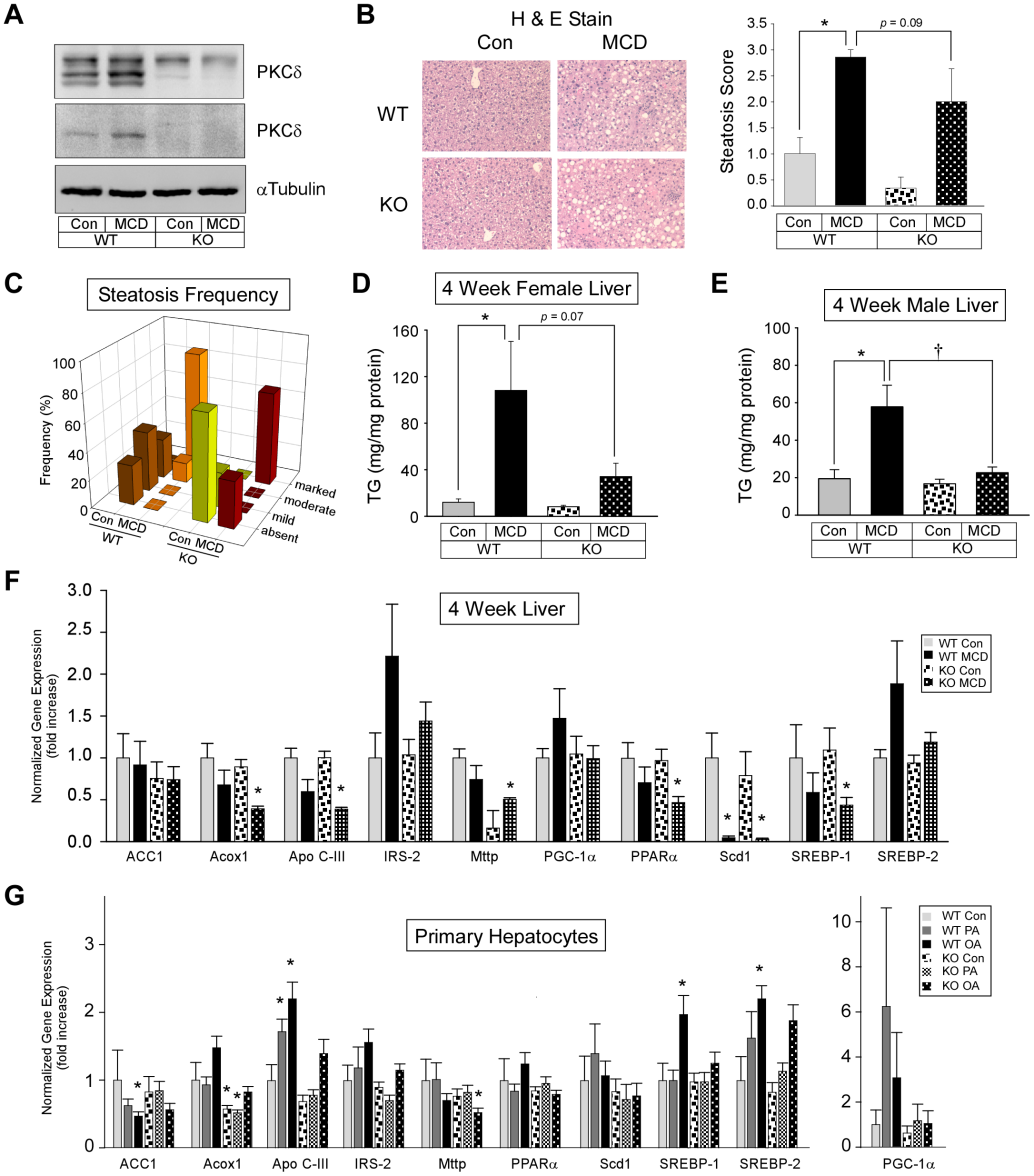
Hepatic PKCδ expression, steatosis, triglyceride accumulation, and lipid metabolism gene expression. (A) Hepatic PKCδ expression using a polyclonal (*upper panel*) and monoclonal (*lower panel*) antibody. PKCδ^+/+^ (WT); PKCδ^−/−^ (KO). (B and D) Hematoxylin and eosin stained liver sections (B) and quantitation shown as the means +/− SE from 6–8 mice per group (D). (C) Quantitation of hepatic triglyceride content is shown as the means +/− SE from 6–8 mice per group. (E and F) Hepatic lipid metabolism relative gene expression of WT and KO mice (n = 6) (E) or BSA (Con), palmitate (PA) or oleate (OA) treated hepatocytes (n = 4) (F). Normalized gene expression and fold change (means +/− SE) relative to Con fed WT mice (E) or WT Con treated hepatocytes (F). (*, p<0.05 versus Con diet fed WT mice or WT Con treated hepatocytes; ^†^, p<0.05 versus MCD diet fed WT mice).

We observed in a prior study that hepatic activation and protein content of PKCδ, but not PKCα or PKCε, is elevated during the development of steatohepatitis in MCD diet fed mice [18]. To investigate the role of PKCδ in the development of steatohepatitis in MCD diet fed mice, PKCδ^−/−^ mice and WT littermates were fed a control or MCD diet for four weeks. As expected PKCδ protein expression was not detected in the liver of PKCδ^−/−^ mice fed a control or MCD diet (Fig. 1A). In agreement with our previous study, hepatic activation and protein content of PKCδ was elevated in MCD diet fed WT mice (data not shown). Consistent with the known effect of MCD diet feeding, WT and PKCδ^−/−^ mice lost body and liver weight, had lower serum levels of glucose and insulin, and higher serum levels of alanine aminotransferase (ALT) (Table 1). However, an ∼50% reduction in serum levels of insulin in control fed PKCδ^−/−^ mice compared to control fed WT mice was observed. Interestingly, MCD diet-induced changes in fat pad weight and fat pad and liver weight normalized to body weight were not observed in PKCδ^−/−^ mice. Also, MCD diet-induced changes in serum triglyceride (TG) and non-esterified fatty acids (NEFA) were not observed in PKCδ^−/−^ mice. In contrast, no difference was observed in MCD diet-induced changes in serum levels of ALT in PKCδ^−/−^ mice. The changes in body and organ weights observed in female WT and PKCδ^−/−^ mice were similar to those in male mice (Table 2), suggesting that the effect of the MCD diet was not sex dependent.

**Table 1:** Weights and serum metabolic parameters in female PKCδ^+/+^ and PKCδ^−/−^ mice.

	PKCδ^+/+^		PKCδ^−/−^	
	Con	MCD	Con	MCD
Body weight (g)	26.7±1.4	15.2±0.5*	21.9±1.9	14.9±0.9*
Glucose (mg/dL)	196±14	100±14*	189±26	100±9*
Liver weight (g)	1.20±0.09	0.68±0.03*	1.02±0.09	0.72±0.08*
Fat pad weight (g)	1.14±0.20	0.15±0.03*	0.59±0.23	0.28±0.23
Liver-body weight (%)	4.48±0.02	4.49±0.19	4.64±0.13	4.80±0.23
Fat pad-body weight (%)	4.12±0.06	0.97±0.19*	2.34±0.76	1.83±1.47
ALT (units/L)	13.7±4.8	139.2±30.4*	8.0±1.9	168.2±20.2*
Triglyceride (mg/dL)	83.7±6.0	57.2±6.3*	67.9±4.7	61.6±4.4
NEFA (mM)	1.63±0.12	1.08±0.05*	1.25±0.15	1.09±0.25
Insulin (ng/ml)	1.50±0.69	0.40±0.05*	0.72±0.15	0.31±0.06*

Con, control diet; MCD, methonine and choline deficient diet; NEFA, non-esterified fatty acids.

Values represent the means ± SEM for n = 5–8.

compared to Con,

p, >0.05.

**Table 2:** Weights and serum metabolic parameters in male PKCδ^+/+^ and PKCδ^−/−^ mice.

	PKCδ^+/+^		PKCδ^−/−^	
	Con	MCD	Con	MCD
Body weight (g)	31.7±1.6	17.5±0.2*	30.3±0.8	18.6±0.8*
Glucose (mg/dL)	194±22	89±5*	173±15	113±4*
Liver weight (g)	1.43±0.09	0.71±0.04*	1.50±0.05	0.82±0.04*
Fat pad weight (g)	1.58±0.30	0.11±0.03*	0.94±0.17	0.21±0.04*
Liver-body weight (%)	4.51±0.16	4.09±0.22	4.96±0.15	4.43±0.16*
Fat pad-body weight (%)	4.85±0.82	0.62±0.16*	3.04±0.50	1.09±0.19*

Con, control diet; MCD, methonine and choline deficient diet; NEFA, non-esterified fatty acids.

Values represent the means ± SEM for n = 5–8.

compared to Con,

p, >0.05.

### Effect of MCD diet on steatosis and lipid metabolism gene expression

Histological examination of livers from mice fed a MCD diet for four weeks showed a 30% reduction in steatosis in PKCδ^−/−^ mice compared to WT mice (*p* = 0.09) (Fig. 1B). When steatosis was examined by frequency of the steatosis score (Fig. 1C) or by Correspondence Analysis (data not shown), the qualitative pattern of steatosis scores from PKCδ^−/−^ mice revealed clear differences compared to WT mice. Consistent with this result, a reduction in liver TG was observed in female and male PKCδ^−/−^ mice (69% and 61%, respectively) compared to WT mice (Fig. 1C and 1D). To determine whether hepatic genes involved in steatosis were differentially regulated in WT and PKCδ^−/−^ mice, we examined the effect of the MCD diet on hepatic genes involved in lipid metabolism. Stearoyl-coenzyme A desaturase 1 (SCD-1) gene expression was significantly decreased in both WT and PKCδ^−/−^ mice (Fig. 1F). However, sterol-regulatory element binding protein-1 (SREBP-1) was significantly decreased only in MCD diet fed PKCδ^−/−^ mice but not WT mice. The expression of peroxisome proliferator-activated receptor α (PPARα), a key regulator of β-oxidation in mice, and its downstream target acyl-coenzyme A oxidase (ACOX1), were significantly reduced in MCD diet fed PKCδ^−/−^ mice but not WT mice. Microsomal triglyceride transfer protein (MTTP) expression in MCD diet fed PKCδ^−/−^ mice was significantly reduced compared to control fed WT mice, yet was induced in MCD diet. Consistent with changes in MTTP expression, a significant reduction in gene expression of apolipoprotein (Apo) C-III, another key determinant of plasma triglyceride metabolism, was observed in MCD diet fed PKCδ^−/−^ mice but not WT mice, suggesting that secreted lipoproteins in PKCδ^−/−^ mice may be more efficiently lipolyzed.

Because differences in steatosis, TG, and hepatic lipid metabolism gene expression were observed between WT and PKCδ^−/−^ mice, we questioned whether fatty acids may have a direct effect on lipid metabolism gene expression independent of a deficiency in methionine and choline. To address this question, we treated primary hepatocytes isolated from WT and PKCδ^−/−^ mice with control or fatty acid containing media and determined lipid metabolism gene expression. Oleate containing medium significantly increased the expression of SREBP-1 and SREBP-2 in hepatocytes from WT mice but not PKCδ^−/−^ mice (Fig. 1G). Although the fatty acids had no effect on SCD-1 gene expression, a significant reduction in ACC1 was observed in oleate treated hepatocytes from WT mice but not PKCδ^−/−^ mice. Palmitate and oleate-induced Apo C-III expression in hepatocytes from WT mice was blocked and blunted, respectively, in hepatocytes from PKCδ^−/−^ mice. A significant reduction was observed in MTTP gene expression in oleate treated hepatocytes isolated from PKCδ^−/−^ mice but not WT mice. Taken together, these results are consistent with the hypothesis that PKCδ plays a role in hepatic lipid metabolism.

### Effect of MCD diet on histological scoring of inflammation and inflammation gene expression

**Figure 2:**
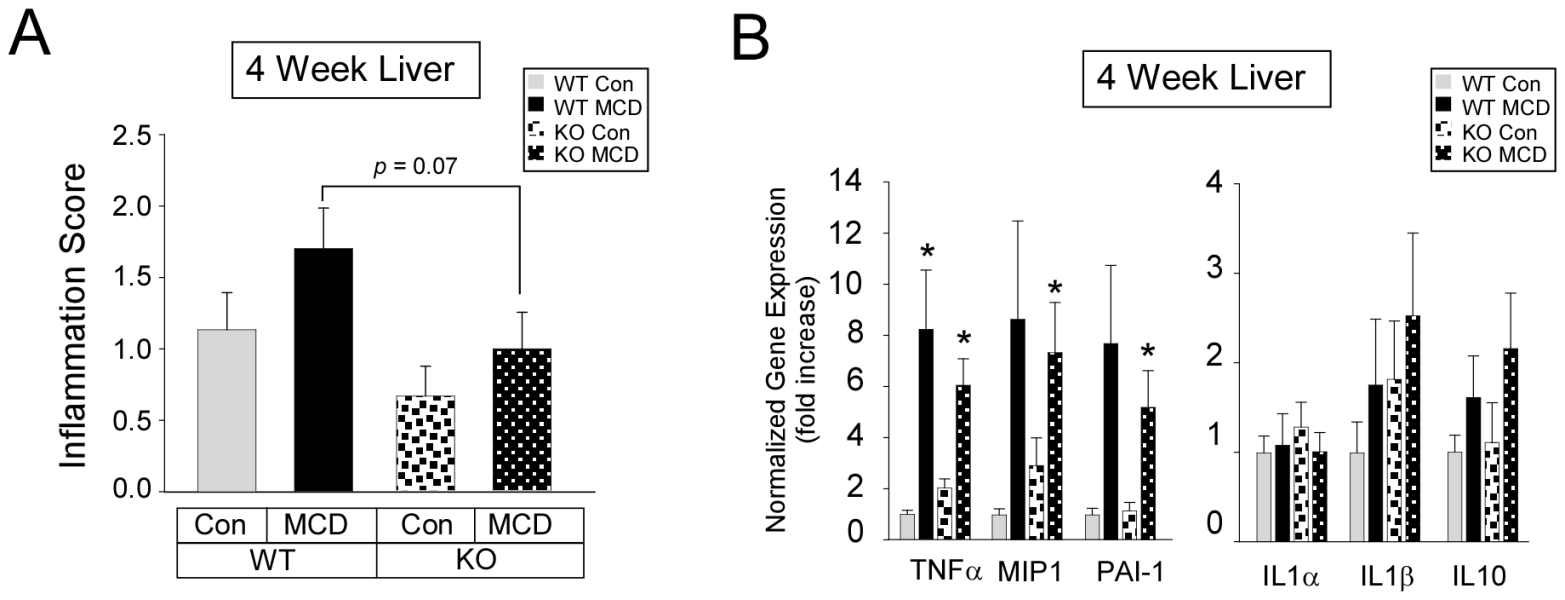
Hepatic inflammation and inflammation gene expression. (A) Scoring of hematoxylin and eosin stained liver sections (means +/− SE) from 6–8 mice per group. (B) Relative expression of hepatic pro and anti-inflammatory genes from Con or MCD diet fed mice (n = 6) for four weeks (means +/− SE). (*, p<0.05 versus Con diet fed WT mice).

Histological examination of livers from mice fed a MCD diet for four weeks showed an approximately 42% reduction in inflammation score in PKCδ^−/−^ mice compared to WT mice (Fig. 2A). However, no differences were found in the hepatic expression of TNFα, macrophage inflammatory protein 1 alpha (MIP1α/CCL3), plasminogen activator inhibitor-1 (PAI-1), interleukin-1 alpha (IL1α), interleukin-1 beta (IL1β), pro-inflammatory genes or an anti-inflammatory gene, interleukin-10 (IL10), from MCD diet fed WT and PKCδ^−/−^ mice (Fig. 2B).

### Effect of MCD diet on markers of oxidative stress and oxidative stress gene expression

**Figure 3:**
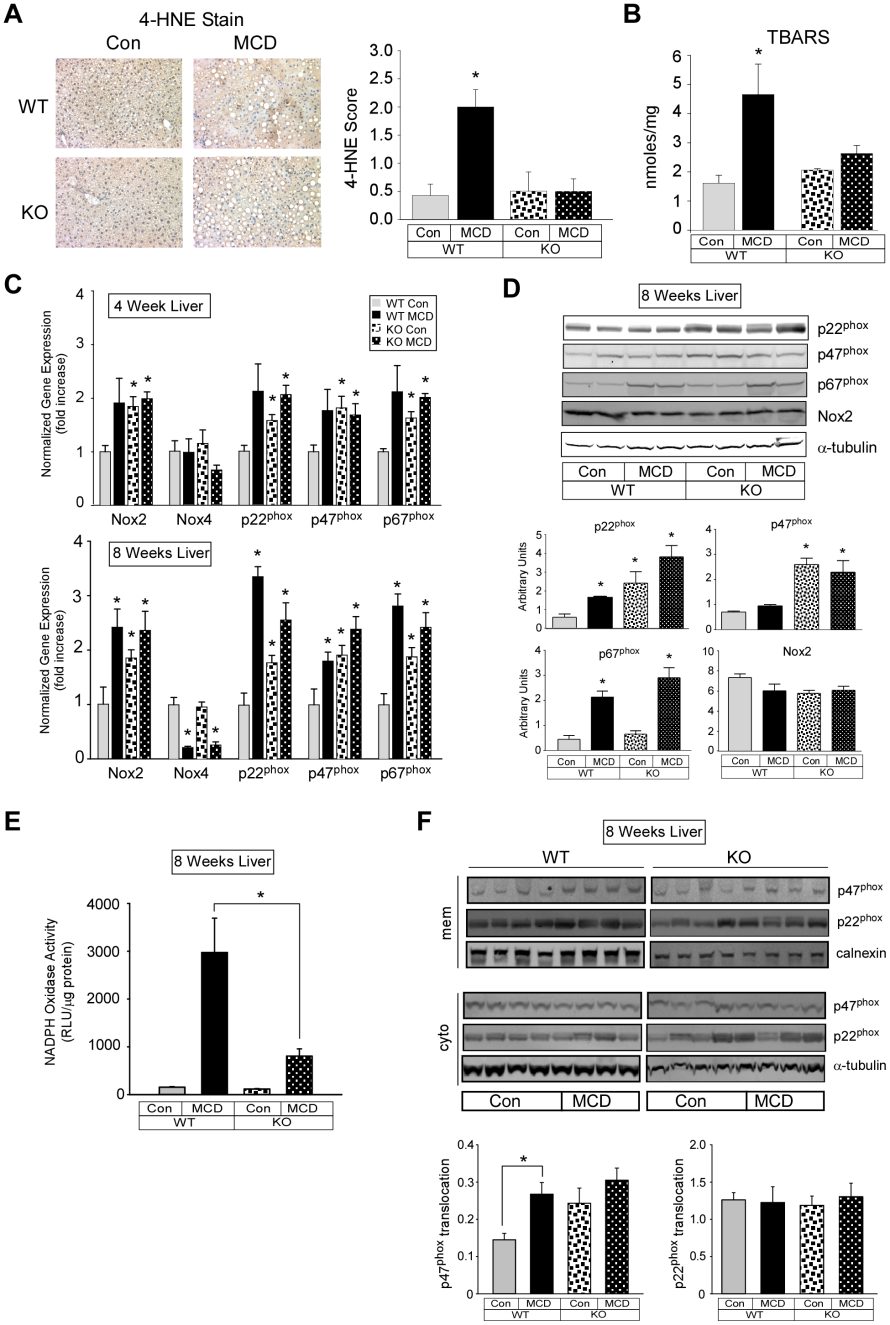
Markers of oxidative stress and oxidative stress gene expression. (A) 4-HNE stained liver sections from four week fed PKCδ^+/+^ (WT) and PKCδ^−/−^ (KO) mice (*left panel*) and quantitation (means +/− SE) (*right panel*). (B) Quantitation of hepatic TBARS (means +/− SE). (C). Relative expression of hepatic NADPH oxidase subunit genes from Con or MCD diet fed mice for four (*upper panel*) or eight (*lower panel*) weeks or hepatocytes treated as in Fig. 1. Normalized gene expression and fold change (means +/− SE) relative to Con fed WT mice. (*, p<0.05 versus Con diet fed WT mice). (D) Hepatic NADPH oxidase subunit protein expression. Total cell lysate (70 µg of protein) from liver tissue was analyzed by immunoblotting for p22^phox^, p47^phox^, p67^phox^, and Nox2 (p91^phox^) and αTubulin expression. Representative immunoblots are shown (*upper panel*) and quantitation of the immunoreactive bands minus background is shown as the means +/− SE (*lower panel*). (*, p<0.05 versus control diet). (E) Hepatic NADPH oxidase activity. Membrane protein (50 µg) was assayed for NADPH oxidase activity as described in the Materials and methods. (*, p<0.05 versus WT). (F). Hepatic NADPH oxidase subunit membrane and cytosolic protein expression. An equivalent amount of membrane and cytosolic protein was analyzed by Western blotting for p22^phox^, p47^phox^, calnexin or αTubulin expression (*upper panels*). Quantitation of the immunoreactive bands minus background of the membrane protein divided by the cytosolic protein (translocation) is shown as the means +/− SE (*lower panels*). (*, p<0.05 versus control diet).

Examination of livers from WT but not PKCδ^−/−^ mice fed a MCD diet showed significantly elevated staining for 4-HNE, a highly reactive aldehyde generated by the exposure of polyunsaturated fatty acids to peroxides and reactive oxygen species (Fig. 3A). Consistent with this result, the MCD diet-induced increase in TBARS in the liver of WT mice was blocked in PKCδ^−/−^ mice (Fig. 3B). Genes involved in the generation of reactive oxygen species in the liver rose 2-fold in four week MCD diet fed WT mice (Fig. 3C, *upper panel*) and were significantly greater than 2-fold by eight weeks in MCD diet fed WT mice (Fig. 3C, *lower panel*). In contrast, a significant reduction in the NADPH oxidase homolog (NOX4) was observed in MCD diet fed WT and PKCδ^−/−^ mice by eight weeks. Surprisingly, expression of NADPH oxidase (NOX2), p22^phox^, p47^phox^, and p67^phox^ were significantly elevated by four and eight weeks in the control diet fed PKCδ^−/−^ mice, while the MCD diet had no additional effect. NADPH expression in the liver at the protein level revealed that NOX2 protein expression was not affected in MCD diet fed WT and PKCδ^−/−^ mice at eight weeks (Fig. 3D). In contrast, p67^phox^ was significantly increased in both WT and PKCδ^−/−^ mice fed the MCD diet for eight weeks. Consistent with the mRNA expression, significantly elevated expression of p22^phox^ and p47^phox^ protein was observed in control diet fed PKCδ^−/−^ mice. To determine the consequence of altered NADPH oxidase subunit expression, we assayed NADPH oxidase activity in the liver from eight week fed MCD mice. NADPH oxidase activity was strongly stimulated in WT mice fed the MCD diet and this activity was significantly reduced in PKCδ^−/−^ mice (Fig. 3E). To gain insight into the mechanism by which MCD diet-stimulated hepatic NADPH oxidase activity was reduced in PKCδ^−/−^ mice, we determined the content of the NADPH oxidase organizing subunit p47^phox^ in membrane and cytosolic protein fractions. Translocation of p47^phox^ from the cytosol to the membrane where it interacts with p22^phox^ leads to NADPH oxidase activation [23]. A significant 84% increase in hepatic p47^phox^ membrane to cytosolic ratio was observed in WT mice fed the MCD diet. In contrast, hepatic p47^phox^ membrane to cytosolic ratio was not significantly increased in PKCδ^−/−^ mice (Fig. 3F). As a control we investigated the membrane to cytosolic ratio of hepatic p22^phox^. As expected, given that p22 is an integral membrane component of NADPH oxidase, no differences in the membrane to cytosolic ratio of hepatic p22^phox^ were observed.

### Hepatic ER stress activation

Expression of IRE1α, Bip/GRP78, phospho-PERK, and phospho-JNK was examined in the livers from four week fed mice (Fig. S1). No effect of the MCD diet was observed on the hepatic protein levels of Bip/GRP78 in WT or PKCδ^−/−^ mice. In contrast, the MCD diet induced elevated levels of IRE1α and phospho-PERK in WT and PKCδ^−/−^ mice. A significant increase in phospho-JNK was observed in WT but not PKCδ^−/−^ mice fed the MCD diet. These data suggest that loss of PKCδ may be involved in MCD diet-induced ER stress mediated by JNK.

### Effect of MCD diet on cell death-cell cycle and apoptosis gene expression

**Figure 4:**
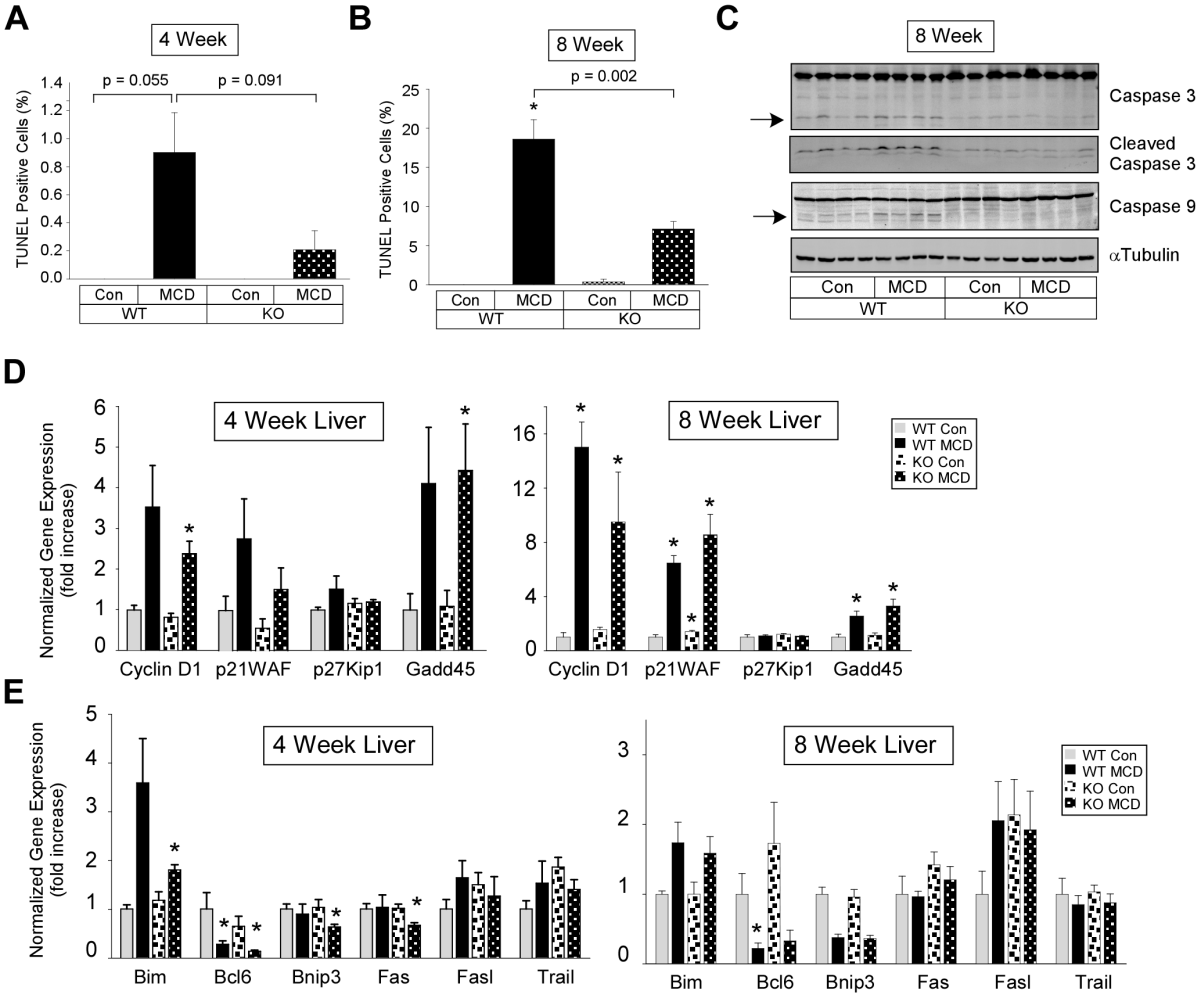
Markers of apoptosis and apoptosis related gene expression. (A) and (B) TUNEL staining of liver sections from four (A) or eight (B) week fed PKCδ^+/+^ (WT) and PKCδ^−/−^ (KO) mice (means +/− SE). (C). Assessment of Caspase cleavage. Total cell lysate (70 µg of protein) from liver tissue was analyzed by immunoblotting for Caspase 9 (mouse specific), Caspase 3, cleaved Caspase 3 (Asp175), and αTubulin expression. Arrows indicate the cleaved 19 and 37 kDa Caspase 3 and 9 fragments, respectively. (D) and (E) Relative expression of hepatic cell cycle (D) and apoptosis (E) genes from four (*left panel*) or eight (*right panel*) weeks fed mice. Normalized gene expression and fold change (means +/− SE) relative to Con fed WT mice. (*, p<0.05 versus Con diet fed WT mice).

Analysis of livers of WT mice fed the MCD diet for four weeks revealed a low number of TUNEL-positive hepatocytes which was reduced in livers from PKCδ^−/−^ mice (Fig. 4A, left panel). In eight week MCD diet fed mice, the number of TUNEL-positive hepatocytes in WT mice was significantly increased to approximately 20%, while less than 10% of hepatocytes in the liver from PKCδ^−/−^ mice were TUNEL-positive (Fig. 4A, right panel). Consistent with these results, MCD diet-induced caspase 3 and 9 cleavage (approximately 36% and 2-fold, respectively) in the livers from WT mice was completely blocked in livers from PKCδ^−/−^ mice (Fig. 4B, right panel).

Analysis of cell cycle gene expression in the liver from WT mice fed the MCD diet for four and eight weeks revealed a significant elevation in Cyclin D1, Gadd45 and p21WAF in eight week fed mice (Fig. 4C). Cyclin D1, p21WAF, and Gadd45 expression was also significantly elevated in eight week fed PKCδ^−/−^ mice. Further analysis of expression for genes regulating apoptosis revealed a significant decrease in Bcl6, Bnip3, and Fas in four week MCD fed PKCδ^−/−^ mice but not WT mice (Fig. 4D, *left panel*). In eight week MCD fed mice, Bcl6 expression was significantly reduced in only the WT mice (Fig. 4D, *right panel*).

### Effect of MCD diet on fibrosis and fibrosis gene expression

**Figure 5:**
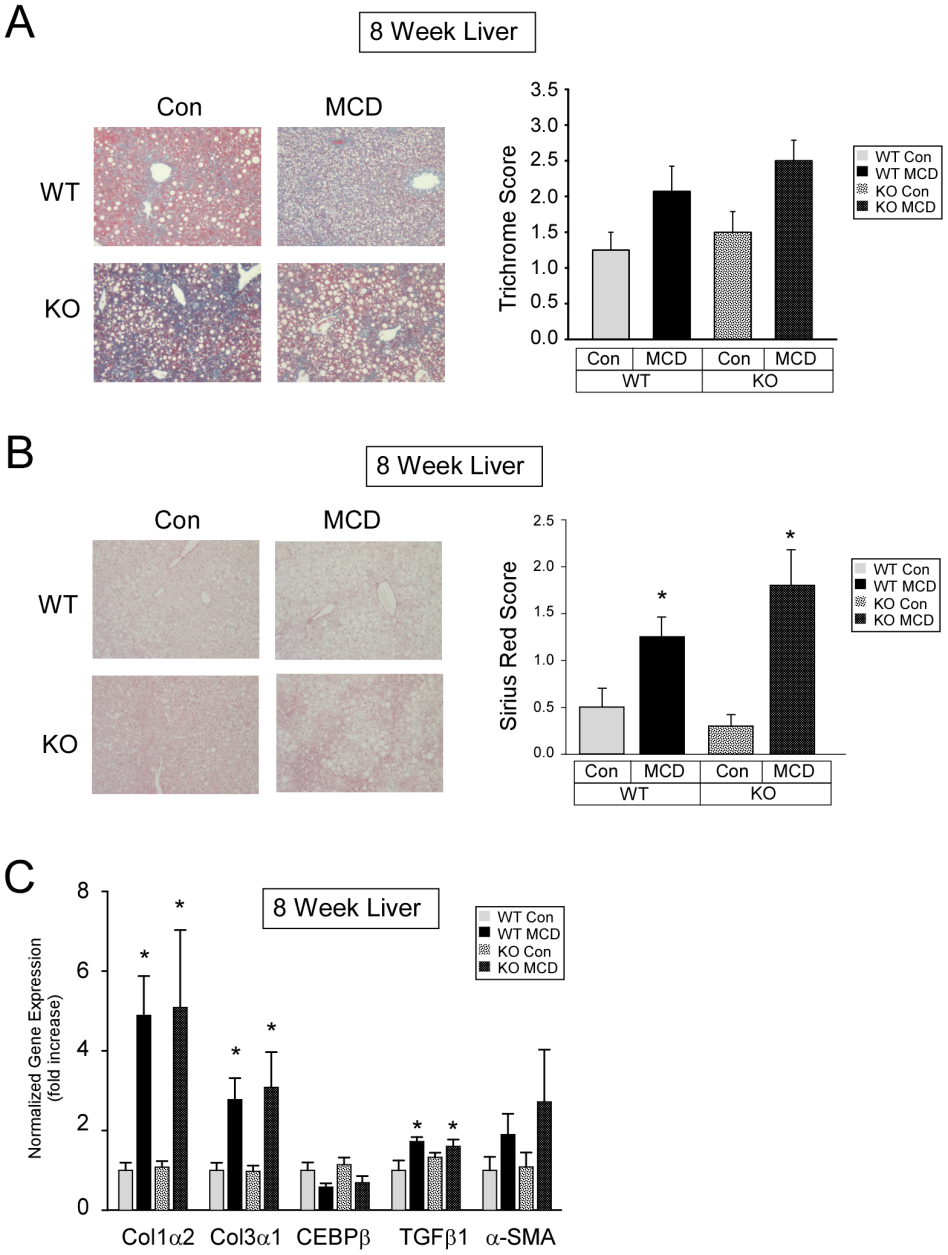
Hepatic Masson's trichrome and Sirius Red staining and fibrosis gene expression. (A) Scoring of Masson's trichrome stained liver sections (means +/− SE). (B) Scoring of Sirius Red stained liver sections (means +/− SE). (C) Relative expression of hepatic fibrosis related genes from Con or MCD diet fed mice for four weeks. Normalized gene expression and fold change (means +/− SE) relative to Con fed WT mice. (*, p<0.05 versus Con diet fed WT mice).

Trichrome and Sirius Red staining of livers from mice fed a MCD diet for eight weeks showed mild to moderate fibrosis in both WT and PKCδ^−/−^ mice (Fig. 5A and 5B). To determine whether the mechanisms by which the MCD diet induced fibrosis were similar in WT and PKCδ^−/−^ mice, we examined the effect of the MCD diet on hepatic genes involved in fibrosis. Collagen (Type IIα2 and Type IIIα1), CCAAT-enhancer-binding protein beta (CEBPβ), transforming growth factor beta (TGFβ), and alpha smooth muscle actin (α-SMA) gene expression were significantly elevated in both MCD diet fed WT and PKCδ^−/−^ mice (Fig. 5C), suggesting that MCD diet-induced fibrosis development was similar in WT and PKCδ^−/−^ mice.

### Effect of MCD diet on PKC isoform gene expression and activation in WT and PKCδ^−/−^ mice

**Figure 6:**
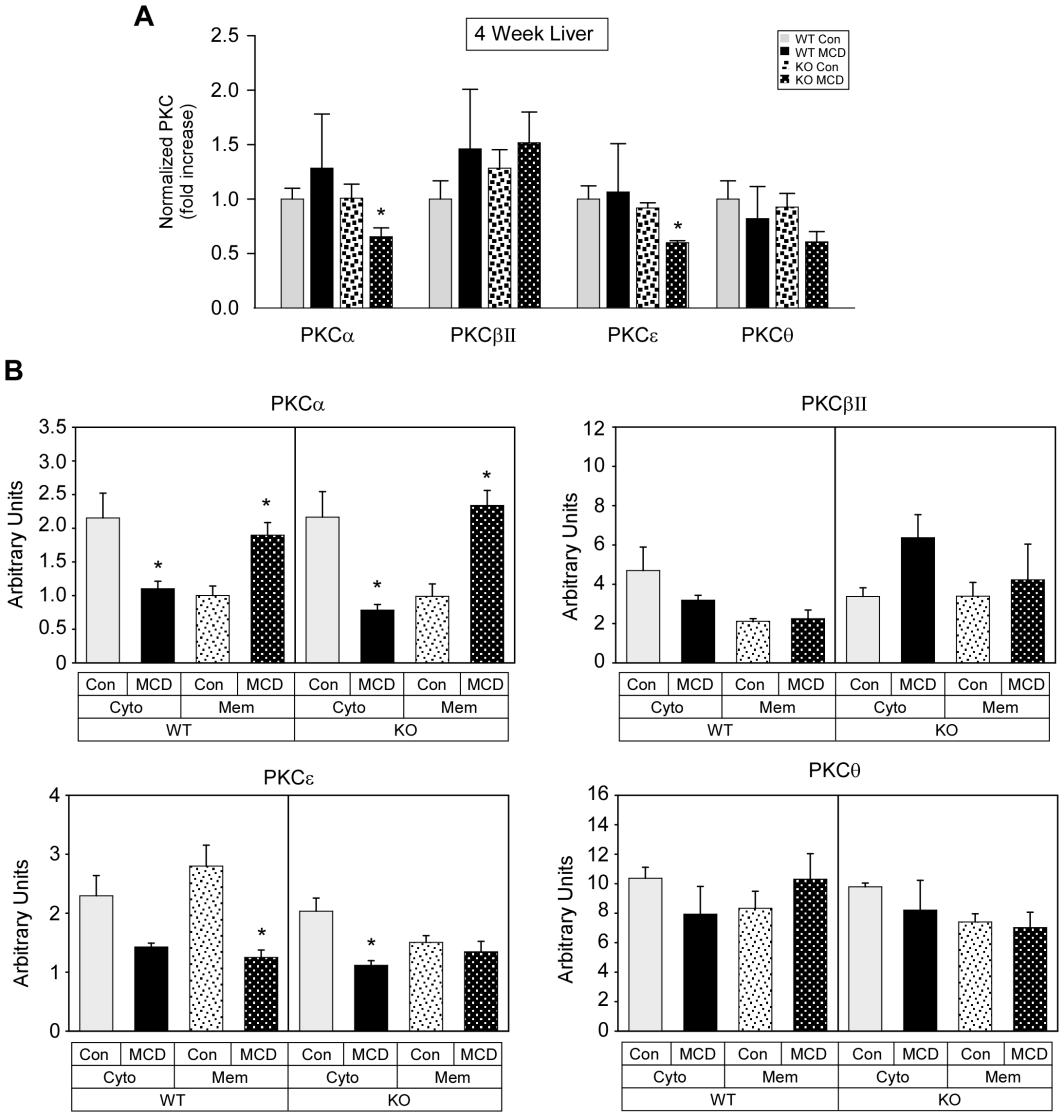
The effect of MCD diet on PKC isoform gene expression and activation in PKCδ^+/+^ and PKCδ^−/−^ mice. (A) PKCα, PKCβII, PKCε, and PKCθ expression was determined in four week fed PKCδ^+/+^ (WT) and PKCδ^−/−^ (KO) mice by quantitative real time PCR and normalized as described in the Materials and Methods. Fold change is shown as the means +/− SE relative to control fed WT mice. (B) An equivalent amount of cytosolic and membrane protein was analyzed by Western blotting for PKCα, PKCβII, PKCε, and PKCθ expression. Quantitation of the immunoreactive bands minus background is shown as the means +/− SE. (*, p<0.05 versus control diet).

To determine whether gene expression of other PKC isoforms was affected in PKCδ^−/−^ mice, relative mRNA levels of PKCα, PKCβII, PKCε, and PKCθ were analyzed in livers from WT and PKCδ^−/−^ mice fed a control or MCD diet. Relative mRNA levels of the PKC isoforms was not affected by the MCD diet in WT mice; however, a significant reduction in PKCα and PKCε mRNA was observed in PKCδ^−/−^ mice (Fig. 6A). Membrane and cytosolic protein content of PKCα, PKCβII, PKCε, and PKCθ was also assessed. Similar changes in PKC isoform membrane and cytosolic protein content were observed in MCD fed WT and PKCδ^−/−^ mice, except for in the MCD fed PKCδ^−/−^ mice where there was an 88% increase in PKCβII cytosolic protein content and a block in the reduction of PKCε membrane protein content (Fig. 6B). Taken together, these results suggest that subtle compensatory changes in the expression or activation of PKC isoforms occur in the liver of PKCδ^−/−^ mice.

## Discussion

Mice fed an MCD diet develop an unusual form of lipodystrophy, weight loss that is accompanied with hepatic steatosis. Hypoglycemia and enhanced whole body insulin sensitivity are additional limitations in the MCD diet model. The weight loss observed in MCD diet fed mice is associated with increased metabolic rate without an increase in food consumption and is associated with suppression in hepatic SCD-1 expression [24]. Indeed, we observed MCD diet-induced weight loss in all mice studied and a profound suppression in hepatic SCD-1 expression in WT and PKCδ^−/−^ mice. MCD diet-induced weight loss is also accompanied by a reduction in fat pad weight relative to body weight which leads to a redistribution of TG stores to the liver. Interestingly, PKCδ^−/−^ mice on the control diet weighed ∼18% less than their WT littermates. The control diet is enriched in sucrose and thus is lipogenic [25]. The reduction in weight gain observed in the PKCδ^−/−^ mice on the control diet is consistent with the results observed by Bezy et. al. with PKCδ^−/−^ mice fed a high fat diet [14].

The mechanism of MCD diet-induced hepatic steatosis is thought to involve an upregulation in the uptake of fatty acids derived from fat coupled with a reduction in the export of VLDL without any major changes to β-oxidation of fatty acids [26]. In the present study the MCD diet-induced reduction in fat pad weight relative to body weight and serum TG and NEFA levels was not observed in PKCδ^−/−^ mice, suggesting that subtle changes to lipid metabolism occurs in PKCδ^−/−^ mice. Consistent with this conclusion, we observed changes in hepatic steatosis and TG and in the expression of genes involved in β-oxidation of fatty acids and VLDL export in four week MCD diet fed PKCδ^−/−^ mice. Consistent with these observations, PKCδ has been shown in high fat fed mice to regulate the induction of hepatic genes involved in lipogenesis [14], [15]. In agreement with these studies, we observed changes in the expression of genes involved in lipogenesis, β-oxidation of fatty acids, and VLDL export in fatty acid treated hepatocytes isolated from PKCδ^−/−^ mice compared to those isolated from WT mice.

A major finding in the present study was the ability of PKCδ to regulate oxidative stress in the liver of MCD diet fed mice. Oxidative stress is a defining characteristic of NASH [27] and is observed in the MCD diet model of steatohepatitis [18]. Our data showed significant reductions in oxidative stress as measured by 4-HNE staining, the detection of TBARS, and a reduction in NADPH oxidase activity in PKCδ^−/−^ mice. These data are consistent with the proposed role of PKCδ in antioxidant induction of defensive mechanisms [28]. Elevated oxidative stress in MCD diet fed mice is associated with the induction of components of the NADPH oxidase complex (Nox2, p22^phox^, p47^phox^, and p67^phox^) [29]. In the present study, we also observed increases in mRNA and protein expression of components of the NADPH oxidase complex in MCD diet fed WT mice. Unexpectedly, we observed significant increases in mRNA expression of Nox2, p22^phox^, p47^phox^, and p67^phox^ and protein expression of p22^phox^ and p47^phox^ in control fed PKCδ^−/−^ mice. Further, the MCD diet was without effect on induction of components of the NADPH oxidase complex in PKCδ^−/−^ mice. These results lead us to speculate that basal oxidative stress is elevated in PKCδ^−/−^ mice, which may play a protective role in the MCD diet fed PKCδ^−/−^ mice.

However, we found that NADPH oxidase activity was not upregulated in PKCδ^−/−^ mice fed the control diet. Thus, the up regulation of NADPH oxidase subunit mRNA and protein in the PKCδ^−/−^ mice does not lead to elevated NADPH oxidase function. To further investigate NADPH activation in MCD diet fed mice, we examined the translocation of p47^phox^, the organizing subunit of the Nox2 NADPH oxidase complex. We observed that MCD diet stimulation of p47^phox^ translocation was impaired in PKCδ^−/−^ mice which is consistent with our data on NADPH oxidase activity. The mechanism by which p47^phox^ translocation is impaired in PKCδ^−/−^ mice is not known. However, PKCδ is one of a number of kinases (e.g. other PKC isoforms, ERK1/2, p38 MAPK, Pak1, and Akt) known to phosphorylate p47^phox^ and regulate translocation [30]. In addition, Nox2 NADPH oxidase activity is modulated by PKC phosphorylation of Nox2 [31] and possibly by phosphorylation of p22^phox^ and p67^phox^ [30].

Apoptosis is a characteristic marker for the progression of steatosis to steatohepatitis. It is associated with an inflammatory response and is thought to play a critical role in the development of fibrosis [32], [33]. The use of caspase inhibitors to reduce the development of NASH and lessen the severity of the MCD diet indicates that apoptosis plays an important role in MCD diet-induced steatohepatitis [34], [35]. We observed a significant increase in hepatocyte apoptosis in mice fed a MCD diet for eight weeks compared to four weeks. Hepatocyte apoptosis was reduced in PKCδ^−/−^ mice compared to WT mice fed a MCD diet. Consistent with the proposed role of caspases in MCD diet-induced hepatocyte apoptosis, we found that caspase activation was blocked in liver of PKCδ^−/−^ mice compared to WT mice. This result is in agreement with other studies linking PKCδ and caspase activation [36]. We did not observe major differences in cell cycle gene expression between WT and PKCδ^−/−^ mice fed a MCD diet. However, a significant reduction in the expression of pro-apoptotic genes in PKCδ^−/−^ mice compared to WT mice fed a MCD diet was observed. These results are consistent with our findings that PKCδ regulates fatty acid-induced cell death in a cellular model of steatohepatitis [18].

In contrast to our findings on apoptosis and caspase activation in the liver, we found that serum ALT, a marker of liver injury, did not differ between WT and PKCδ^−/−^ mice fed a MCD diet. Thus, a lack of congruency was observed between serum ALT levels and oxidative stress and apoptosis in MCD diet fed PKCδ^−/−^ mice. A lack of congruency in serum ALT levels in MCD diet fed mice has also been observed by others. For example, a complete block in MCD diet-induced hepatic NADPH oxidase activity has been observed in the presence of significantly elevated serum ALT levels are in TLR4^−/−^ mice [29], while a lack of congruency between serum ALT and proinflammatory gene expression and development of fibrosis has been observed in caspase-1^−/−^ MCD diet fed mice [37]. Finally, a recent study in NAFLD patients observed a lack of correlation between serum liver enzymes including ALT and changes in steatosis, inflammation, hepatocyte ballooning, or fibrosis stage over time [38].

Our observation that gene expression of TNFα, MIP1α/CCL3, PAI-1, IL1α, and IL1β which have been shown to be upregulated in the liver of MCD diet fed rodents [39]–[41] did not differ in the liver between PKCδ^−/−^ and WT mice, even though a reduction in histological inflammation score in PKCδ^−/−^ mice compared to WT mice was observed, suggests that PKCδ plays a role in steatohepatitis progression downstream from the induction of inflammation. Consistent with this conclusion are results demonstrating that TNFα and MCP-1 activate PKCδ [20], [42] and that lipid-induced PKCδ activation in the liver is associated with increases in serum levels of MCP-1 [9]. Alternatively, the uncoupling of elevated proinflamatory gene expression and a reduction in histological inflammation score in PKCδ^−/−^ mice compared to WT mice could be due to differential polarization of resident macrophages (Kupffer cells) or infiltrated macrophages [43] and/or changes to immune cell dynamics in the liver [44].

A major surprise in our study was that no difference in Masson's trichrome and Sirius Red staining of liver sections was observed between WT and PKCδ^−/−^ mice fed the MCD diet for eight weeks. Consistent with our staining results, we observed no differences in the expression of hepatic fibrosis genes known to be upregulated in MCD diet fed mice [45]. Thus, the results from our model indicate that reducing hepatic inflammation and oxidative stress does not ameliorate hepatic fibrosis development. However, it possible that the degree of inflammation and oxidative stress reduction we observed may have led to a reduction in fibrosis at a later time point in the MCD diet feeding.

Our results do not agree with the recent study by Lee et al. [46] in which rottlerin and a PKCδ short peptide inhibitor blocked fibrogenic gene expression and fibrosis in mice fed a MCD diet for 3 weeks and treated with LPS for 6 h. However, the LPS treatment lead to significant increases (from 8- to 15-fold) in hepatic TGF-β, α-SMA, and pro-collagen-1 α1 mRNA expression compared to that observed in the untreated, MCD diet fed mice. Further, Masson's trichrome staining was observed in the 6 h LPS treated, MCD diet fed mice but not in the untreated, MCD diet fed mice. Thus, fibrosis development appears to be an acute response in the LPS-treated, MCD fed mice compared to fibrosis development in the present study which was observed in mice fed the MCD diet for 8 weeks. Finally, the LPS independent effects of PKCδ on key pathophysiological features of NASH are not known in the LPS-treated, MCD diet model of NASH. Further, given that there are key pathophysiological features in human patients with NASH (obesity and whole body insulin resistance) which are not present in the MCD diet model of NASH, it is possible that PKCδ deletion in the context of obesity and whole body insulin resistance may reduce fibrosis.

A limitation in our study is that our findings are based upon a whole body knockout of PKCδ. The development of NASH is a complex pathophysiological process that requires cross-talk between the liver and other major organ systems such as adipose tissue [47]. Thus, the role of PKCδ in key aspects of the pathophysiology of NASH in the liver and adipose tissue will require tissue-specific knockout models.

In conclusion, we have shown that PKCδ regulates lipid metabolism, oxidative stress, and apoptosis, key aspects of the pathophysiology of MCD diet induced steatohepatitits in mice. Our key finding that PKCδ regulates apoptosis in MCD diet fed mice suggest that our results may have relevance to the human condition where apoptosis is being targeted to treat NASH [48]. In addition, our studies using primary hepatocytes suggest that PKCδ modulates the direct effect of fatty acids on lipid metabolism expression. Finally, the data presented here suggest that increased PKCδ protein levels and activation may be involved in the development of NASH.

## Supporting Information

Figure S1 **Hepatic ER stress activation.** Liver tissue from mice on control or MCD diets for four weeks was pulverized under liquid N_2_, and lysed in detergent containing buffer. A. Total cell lysate (60 µg of protein) from liver tissue was analyzed by immunoblotting for IRE1, phospho-PERK, phospho-JNK, BiP, and αTubulin expression. B. Quantitation of the immunoreactive bands minus background is shown as the means +/− SE (*, p<0.05 versus control diet).
(TIF)
